# Combined neutralization of interferon gamma and tumor necrosis factor alpha induces IL-4 production but has no direct additive impact on parasite burden in splenic cultures of human visceral leishmaniasis

**DOI:** 10.1371/journal.pone.0199817

**Published:** 2018-06-28

**Authors:** Neetu Singh, Shyam Sundar

**Affiliations:** Department of Medicine, Institute of Medical Sciences, Banaras Hindu University, Varanasi, Uttar Pradesh, India; Pasteur Institute of Iran, ISLAMIC REPUBLIC OF IRAN

## Abstract

Immune activating cytokines Interferon (IFN)-γ and Tumor necrosis factor (TNF)-α are known to activate macrophages for killing of Leishmania parasite. IFN-γ provides therapeutic potential while TNF-α has been recognized to mediate protection in visceral model of infection. In the present study we investigated whether combination of IFN-γ and TNF-α has better therapeutic strength than individually using one of these cytokines in Visceral Leishmaniasis (VL) patients. We performed combined blockade of IFN-γ and TNF-α in VL splenic biopsies and demonstrated it’s impact on number of viable amastigotes and cytokine production. Additionally, selective depletion of splenic cell subsets was performed to establish the cellular sources of IFN-γ and TNF-α. Treatment of splenic aspirate cells with combination of anti-IFN-γ and anti-TNF-α monoclonal antibodies for 72 hours enabled no direct additive impact of these cytokines on parasite replication and IL-10 secretion, but IL-4 production was induced. Further assessment of splenic biopsies put forward CD4^+^ T cells as a source of IFN-γ whereas CD14^+^ cells contribute towards TNF-α production. Overall our results suggest, the interplay of pro-inflammatory cytokines IFN-γ derived from CD4^+^T lymphocytes and TNF-α from CD14^+^ cells has no direct additive impact on parasite replication but induces IL-4 production. Our data does not support direct targeting of IFN-γ and TNF-α for combination therapy but targeting these cytokines as an adjuvant in patients with exaggerated tissue inflammatory responses can have favourable patient outcome.

## Introduction

Visceral Leishmaniasis (VL) is a principal public health problem in developing countries and is amongst the utmost neglected tropical diseases [1]. Majority of VL infection remains asymptomatic and the mechanisms through which parasite are cleared remains unknown [2]. In the absence of human vaccine favoured treatment option for VL remains chemotherapy, which is further limited by drug toxicity, unresponsiveness and development of resistance [3]. Host immune modulation therapies has been suggested as a strategy to further improve the efficacy of drug treatment [4]. Immune activating cytokines are recognized to strengthen host defence by curbing intracellular parasite [5] and on that account can enhance chemotherapeutic efficacy.

IFN-γ plays a particular role of macrophage activation for anti-leishmanial activity [6] and IFN-γ blockade causes significant increase in number of viable amastigotes in splenic aspirate cultures from VL [7]. Moreover, IFN-γ has been suggested as an adjunct to conventional antimonial treatment [8–10]. Similarly, TNF-α is a pro-inflammatory cytokine and has been implicated to mediate resistance against *L*. *donovani* infection in a visceral model of infection [11–13]. However, TNF-α neutralization in VL splenic cultures does not affect the parasite burden [14]. In murine models, IFN-γ and TNF-α are believed to act in synergy for elimination of parasite [15], through induction of nitric oxide (NO) in macrophages to kill intracellular *Leishmania major* [15, 16]. But, direct effect of IFN-γ and TNF-α on parasite burden and cytokine production remains unclear in human VL. This highlights the need to undertake a human study in clinical setting, to determine whether combination of IFN-γ and TNF-α works better for parasite elimination than their individual usage.

Present study demonstrates the interaction between IFN-γ and TNF-α with a view towards development of understandings if this would be a better target for immune modulation therapies in VL. Splenic biopsy from patients comprises infected macrophages, effector cells and suppressive lymphocytes and as such culture of splenic aspirate require little manipulation of cells hence offers robust assay to monitor effect of cytokine based modulation. Here, we established the splenic cellular source of IFN-γ, TNF-α and demonstrated their combined impact on parasite load and cytokine responses.

## Materials and methods

### Study subjects

All patients recruited in the study were presented with typical symptoms of VL and seeking treatment at the Kala-azar Medical Research Centre (KAMRC), Muzaffarpur, Bihar, India. In total, 42 untreated VL cases with a confirmed diagnosis were included in the study. VL is confirmed by detection of amastigotes in splenic aspirate smears and/or antibodies against recombinant (r) -K39 antigen using commercially available dipstick strip (InBios Kala Azar Detect Rapid Test, USA). The aggregate clinical features of all the enrolled cases are described in Table 1. Only those subjects who agreed to participate and are above 14 years were enrolled in the study. Patients previously detected with kala-azar or positive for HIV, hepatitis, tuberculosis, malaria, unable to provide informed assent or pregnant females were not included in the study. None of the donor has received any anti-inflammatory medication prior to sample collection. All patients were treated with Amphotericin B in either its liposomal ordeoxycholate form for 30 days [17] and were completely recovered following treatment.

**Table 1:** Aggregate clinical features of all the VL patients enrolled in the study.

Variables	VL patients (n = 42)
Age (years)	34.71 ± 13.61 (33)^a^
Sex (M/F)	28/14
Splenic score ^b^^,^^c^	2.19 ± 1.04 (2)
Splenic enlargement (cm) (Pre-treatment)	4.73 ± 2.80 (4.5)
Splenic enlargement (cm) (Post-treatment)	0.5 ± 1.04 (0)
Haemoglobin (g/dL) (Pre-treatment)	8.10 ± 2.15 (7.7)
Haemoglobin (g/dL) (Post-treatment)	10.25 ± 1.43 (10.25)
Duration of illness (days)	46.57 ± 63.30 (21.5)
Maximum fever (°F) (Pre-treatment)	99.82 ± 8.86 (101)
WBC count (per mm^3^) Pre-treatment	3440.47619 ± 2116.8383 (3050)
WBC count (per mm^3^) Post-treatment	6985.7142 ± 2735.8123 (6350)
Platelet count (per mm^3^) (Pre-treatment)	96142.85 ± 69743.63 (82500)
Platelet count (per mm^3^) (Post-treatment)	209023.8095 ± 73487.5095 (206500)
Lymphocytes (%) (Pre-treatment)	49.78 ± 13.37 (50)
Lymphocytes (%) (Post-treatment)	37.28 ± 9.85 (36)
Neutrophils (%) (Pre-treatment)	49.09 ± 10.66 (49.5)
Neutrophils (%) (Post-treatment)	63.02 ± 9.07 (63.5)
Monocytes (%) (Pre-treatment)	1.59 ± 0.66 (1.5)
Monocytes (%) (Post-treatment)	1.4 ± 0.59 (1)

^a^ Median values are given within parenthesis

^b^ Parasite scoring is on logarithmic scale from 0 to 6, where 0 stands for no parasite in 1000 microscopic fields (1000X), 1 is 1–10 parasites per 1000 fields and 6 is > 100 parasites per field.

^c^ Splenic scores presented are based on patients undergone biopsy

### Ethics statement

The use of human subjects obeyed recommendations listed in the Helsinki declaration. Prior informed written consent was obtained from participants or their legal guardian, in case of minor. Standardized questionnaire was used to record clinical and demographical characteristics of the participants. Institutional ethical approval (IRB No. Dean/2008-09/314, Dean/2012-2013/89) was obtained from the ethical review board of Banaras Hindu University (BHU), Varanasi, India.

### Collection of splenic aspirates

Splenic aspirate (SA) samples were collected at the KAMRC and splenic biopsy was conducted before initiation of VL treatment for parasitological confirmation of disease. Only the residual splenic material, left after the smear preparation for diagnostic purposes is used and in no case biopsies has been obtained solely for the purpose of study. The left over SA material (50–150 μl, 1–3 million cells) was available for research and is sufficient to carry out our assays. Needles with SA were flushed out into 0.9mL heparinised RPMI 1640 media (Gibco, USA) supplemented with 10% heat inactivated (HI)—FBS (Gibco), 50 Units/ml Penicillin, 50μg/ml Streptomycin (Gibco), 25mM HEPES (Gibco). These splenic suspension samples were transported at 4–8°C from the KAMRC hospital to the central lab at BHU and immediately processed upon arrival. Splenic suspension cells were submitted to ex-vivo short term culture assay for analyses of secreted cytokines and parasite growth.

### Culture of splenic cells

#### Anti-cytokine treatment in SA culture

**Fig 1:**
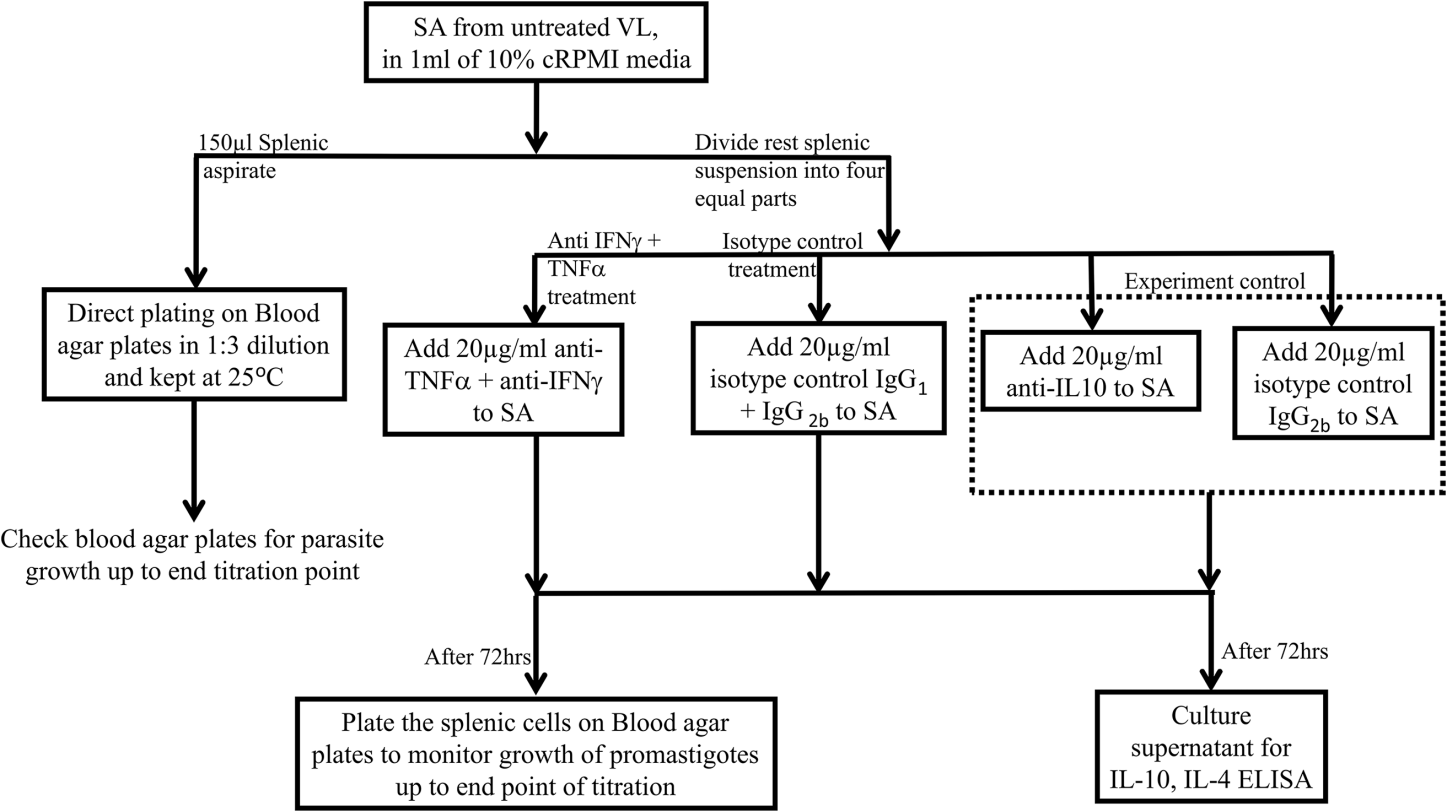
Stepwise representation of various procedures involved in the neutralisation and short term splenic cell culture assay on VL subjects.

A small fraction of SA suspension (150μl) was directly inoculated into biphasic medium of blood agar plates overlaid with 100μl of 20% cM199 media for baseline quantification of amastigotes employing limiting dilution method, as described elsewhere [18, 19]. The remaining splenic material was seeded into four equal parts (200 μl per well) in U-bottom 96-well polypropylene culture plate (Nunc, Roskilde, Denmark). Mouse monoclonal antibodies against TNF-α (clone 1825, R&D Systems, USA) in combination with IFN-γ (clone 25723, R&D Systems, USA) or control immunoglobulin IgG_1_ (clone 20116, R&D Systems, USA) together with IgG_2B_ (clone 20116, R&D Systems, USA) were added. Previously, we showed that IL-10 blockade in SA promotes parasite clearance [20]. Therefore, as an experimental control for our blocking assay we cultured SA in presence of anti-IL-10 (clone 25209, R&D Systems, USA) antibody and it’s isotype control IgG_2B_ (clone 20116, R&D Systems, USA). All monoclonal antibodies were used at a final concentration of 20μg/ml. The splenic cells were incubated with neutralising antibodies for 72 hrs at 37°C in an atmosphere of 5% CO_2_ in air. Following three days of incubation culture supernatants were removed and stored at −80°C for cytokine measurement. The removed culture volume was replaced by M199 medium, supplemented with 20% FBS, 50μg/ml streptomycin, 50U/ml penicillin, 40mM HEPES, 0.1mM adenine (all media components from Gibco, USA). Thenceforth, SA cells were transferred into blood agar plates for titration culture, as described above. Fig 1 demonstrates stepwise procedures involved in the neutralisation and short term splenic cell culture assay.

#### Ex-vivo quantitation of viable tissue amastigotes within SA

Number of viable splenic tissue amastigotes present in culture and cytokines produced in supernatant were used as a read out to demonstrate the impact of cytokine modulation. Viable amastigotes were quantified by performing a serial dilution, as described elsewhere [19, 21, 22]. Micro titration approach is preferred over qPCR to ensure quantification of viable *Leishmania* parasite. Briefly, each SA was diluted threefold in 96-well blood agar microtiter plates over twelve wells. *Leishmania* parasites from splenic cultures were grown out on blood agar plates, overlaid with M199 medium supplemented with 20% FBS. The culture plates were routinely monitored for parasite growth following 7–10 days of incubation at 25°C in a BOD incubator. The number of viable parasites was determined by the highest dilution or last well up to which promastigotes could be grown.

### Depletion and culture of splenic biopsies

To demonstrate the splenic cellular sources of IFN-γ and TNF-α, selective depletion of cell subsets was performed and impact of cell removal would be manifested on ex-vivo cytokine production. Cells were gently depleted from SA employing super paramagnetic Whole blood Micro beads (Miltenyi Biotec, Germany) and MACS Column technology which is specially designed for positive selection of cell subsets directly from anti-coagulated samples. Whole blood Micro beads against CD14 (Cat No. 130-090-879) and CD4 (Cat No. 130-090-877) were used for magnetic labelling of cells in 0.5mL of splenic suspension as per manufacturer’s instruction. Briefly in separate set of experiment, fifteen splenic suspension was divided into two equal parts (500 μl), either treated with CD4 (n = 6)/CD14 (n = 9) Micro beads or with FITC beads, to control the effect of spontaneous bead uptake. Further magnetically labelled cell suspension was applied onto the prepared Whole blood column. Magnetically labelled cells are retained in the column while flow through containing unlabelled cells passes via column and collected in separate tubes for subsequent culture in round bottom polypropylene tubes at 37°C in 5% CO_2_ for 24hrs. Following incubation splenic culture supernatant was collected for cytokine estimation by ELISA.

### Cytokines measurements

Commercially available kits ELISA Max Deluxe Set Human IL-10 (Cat No. 430605), IFN-γ (Cat No. 430105), TNF-α (Cat No. 430204) and IL-4 (Cat No. 430304) (Biolegend, U.S.A) were used to estimate concentration of cytokines in splenic culture supernatants, using the manufacturer’s instructions. The manufacturer-reported detection sensitivities for ELISA MAX Deluxe Set kits are 2 pg/mL for IL-10, TNF-α and IL-4 whereas 4 pg/mL for IFN-γ cytokine.

### Statistical analyses

Comparison between two groups was performed employing either Mann Whitney Test or Wilcoxon matched pair test. All statistical analyses were performed using PRISM7 (GraphPad, La Jolla, CA, USA) software and the effect of antibody treatment was considered significant when p<0.05.

## Results

### Effect of anti-IFN-γ and anti-TNF-α treatment on number of viable *Leishmania* parasite in SA cultures

**Fig 2:**
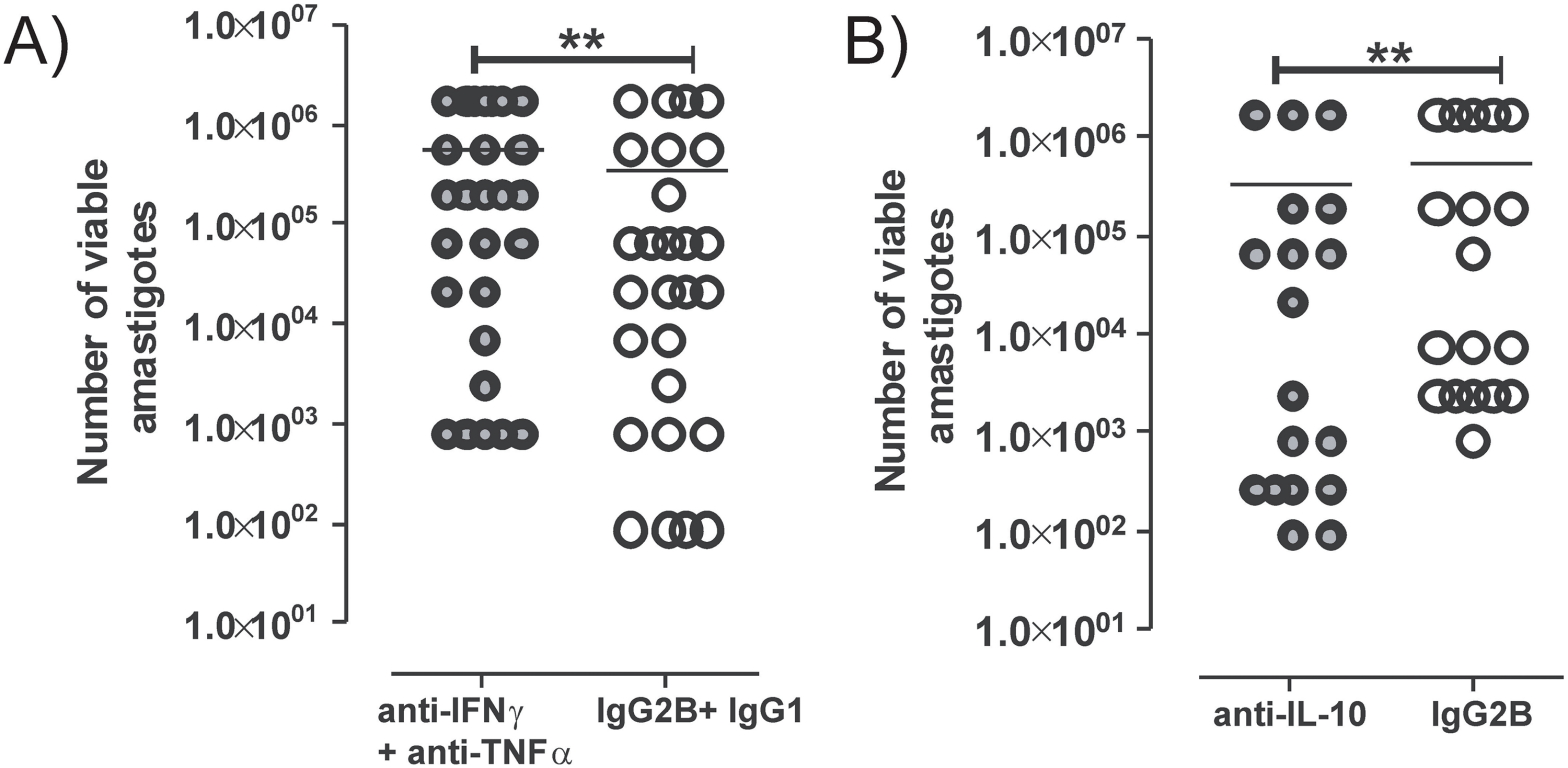
Effect of anti-IFN-γ + anti-TNF-α antibody on number of viable amastigotes. Splenic biopsies were treated with monoclonal neutralising antibodies in a 72hr culture. Following three days of incubation number of viable parasite was determined using titration culture. Results obtained were presented on vertical scatter dot plot **(A)** Splenic cultures (n = 27) were plated after treatment with neutralising antibodies against human IFN-γ and TNF-α (solid grey circles) or with their isotype controls (open circles). **(B)** Apart of splenic biopsy (n = 18) was also treated with anti-IL-10 monoclonal antibody (solid grey circles) and its isotype control IgG_2B_ (open circles), as a benchmark for this neutralising assays in splenic cells. Number of viable amastigotes in cultures is presented on log scale and in scientific format. Antibody treated samples were compared with their corresponding isotype control treated cultures using Wilcoxon matched pair test. Statistical significances are shown with a P value **P<0.01.

We performed an ex-vivo combined neutralisation of IFN-γ and TNF-α, to investigate their integrated impact on parasite growth and cytokine production. Treatment of splenic cells with anti-IFN-γ and anti-TNF-α monoclonal neutralizing antibodies enabled increase in the number of viable tissue amastigotes, conversely these cytokines mediate parasite killing. This confirms the activating role of these cytokines in VL spleen. SA biopsies treated with combination of anti-IFN-γ mAb and anti-TNF-α mAb display significant increase in parasite burden (**P = 0.01) in 59% of SA cultures (16/27) while no changes in 26% of cultures were reported (7/27) and 14.8% (4/27) SA showed decrease in parasite numbers (Fig 2A).

### IL-10 blockade in SA cultures

Previously we showed, IL-10 neutralization promotes parasite clearance in SA biopsies from VL patients [20]. To provide benchmark for our combined blockade cell culture assay we performed neutralisation of cytokine IL-10 using monoclonal antibody in 72hr culture, and demonstrated the impact of IL-10 blockade on parasite burden. As shown in Fig 2B and in line with our previous report, we found a significant decrease in the number of viable amastigotes in 83% of anti-IL-10 mAb-treated SA cultures tested, in comparison to splenic cultures treated with anti-IgG_2B_ isotype control mAb (Fig 2B). This confirms the deactivating role of IL-10 in VL spleen.

### Combined blockade of IFN-γ and TNF-α increases IL-4 production but does not alters IL-10 secretion in SA cultures

**Fig 3:**
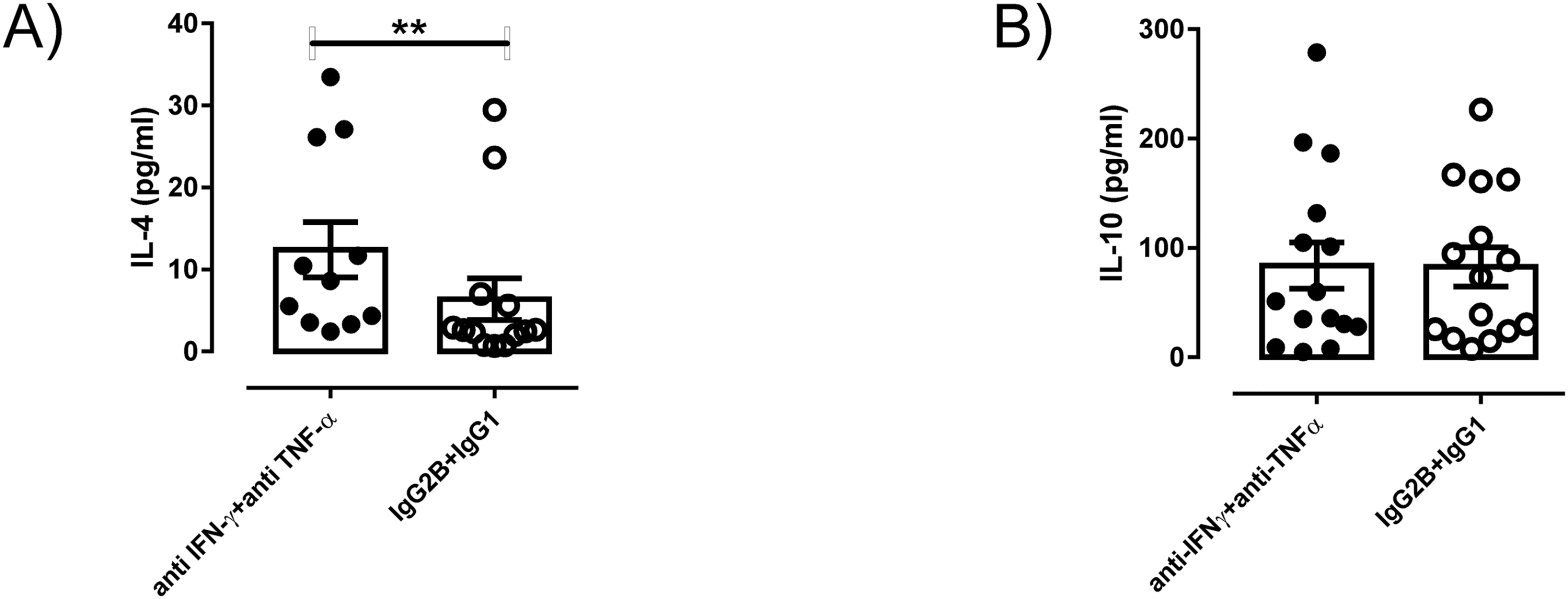
Effect of anti-IFN-γ + anti-TNF-α antibody on cytokine production in splenic aspirate cultures of human VL. Splenic supernatants from anti-IFN-γ + anti-TNF-α antibody treated cultures were used for estimation of cytokines employing ELISA. Value shown **(A)** IL-4 (pg/ml) and **(B)** IL-10 (pg/ml) are cytokine concentrations. Statistical analyses were executed using Wilcoxon matched-pair test. Solid black dots represent splenic aspirate cells devoid of CD4^+^ or CD14^+^ subset whereas open circles show splenic biopsy treated with control magnetic beads.

Further, we tested whether combined neutralization of IFN-γ and TNF-α influenced type II cytokine production during VL, by assessing the concentrations of IL-10 and IL-4 in SA culture supernatants. Combined neutralization of cytokines IFN-γ and TNF-α resulted in a significant increase in IL-4 levels in 70% of samples tested, compared to isotype control treated cultures (Fig 3A). However, no significant changes in IL-10 levels were reported in these same SA culture supernatants (Fig 3B). Wide range of estimated cytokine concentrations show variability in number of cells plated.

### Cellular source of IFN-γ and TNF-α in VL spleen

**Fig 4:**
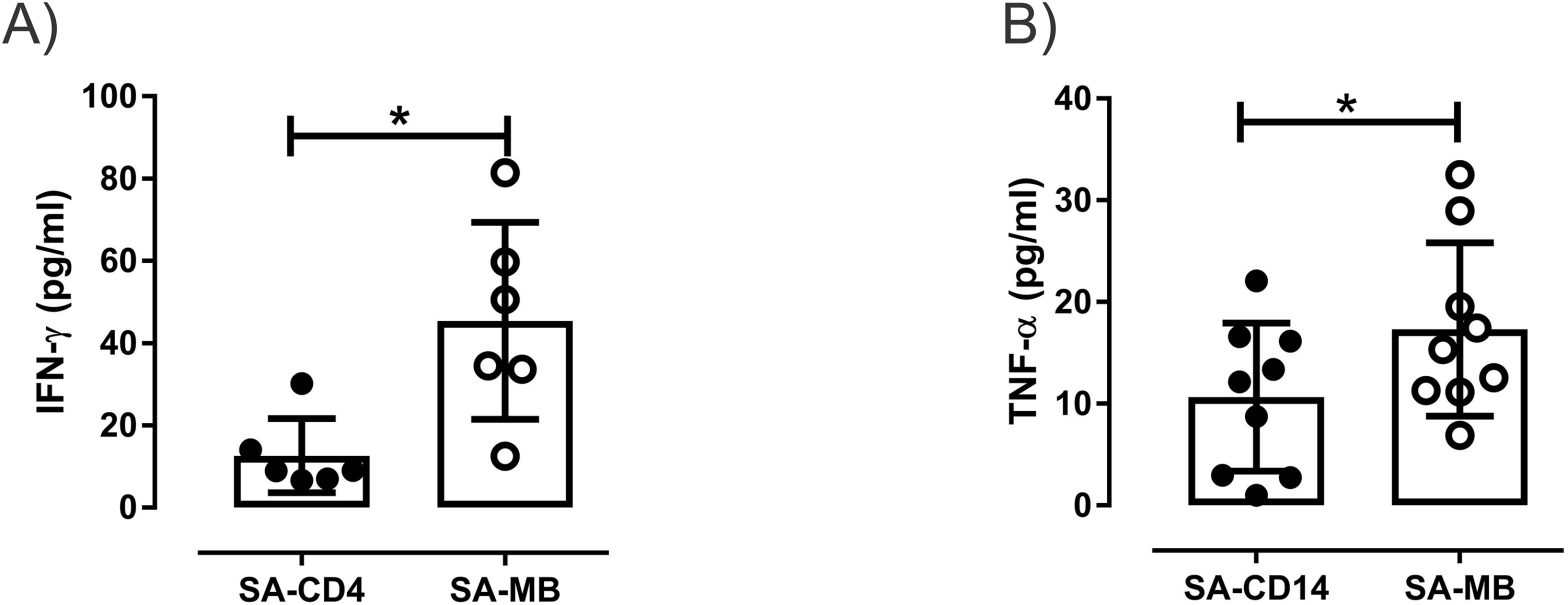
Splenic cellular source of IFN-γ and TNF-α in VL. Vertical scatter dot plots show concentration of cytokines **(A)** IFN-γ (pg/ml) and **(B)** TNF-α (pg/ml) in splenic culture supernatants following 24hrs of incubation at 37°C in the atmosphere of 5% CO2, after depletion of CD4^+^ (n = 6) and CD14^+^ (n = 9) cells respectively, using whole blood micro beads from splenic biopsy of untreated VL. Statistical analyses were executed using Wilcoxon matched-pair signed rank test. Solid black dots represent splenic aspirate cells devoid of CD4^+^ or CD14^+^ subset whereas open circles show splenic biopsy treated with control magnetic beads.

To demonstrate the splenic cellular sources of IFN-γ and TNF-α, we analysed the effect of specific cell removal from SA on ex-vivo cytokine production. Cytokine ELISA from these culture supernatants suggest significant loss in IFN-γ production from the SA cultures devoid of CD4^+^ T cells in comparison to control micro bead treated cultures (*P = 0.03) (Fig 4A). Additionally, splenic cultures depleted of CD14^+^cells (monocytes-macrophages) lowers TNF-α secretion in the supernatant, when compared with controls (*P = 0.01) (Fig 4B). These data suggest involvement of CD4^+^ T cells and CD14^+^ cells in the production of endogenous IFN-γ and TNF-α in VL spleen.

## Discussion

Therapeutic efficacy of IFN-γ and TNF-α is emulated in their capability to activate macrophages for intracellular parasite killing [15]. Here, we used a well-established ex-vivo splenic aspirate culture assay to address how these immune activating cytokines interact in the target organ of VL. Our attempt to assess the interplay of IFN-γ produced by CD4^+^cells and TNF-α secreted through CD14^+^ cells revealed no direct additional impact of these cytokines on parasite survival and IL-10 production but an enhanced release of IL-4 was reported by splenic cells during the 72hr culture.

Direct titration of splenic biopsies revealed wide range of viable amastigotes present in aspirate. This variability further reflects sampling variance involved in needle aspiration process such as concentration or volume of biopsy besides stage of disease. Further in 26% of neutralised cultures for whom no change in parasite numbers was observed, did not differ significantly in any clinical feature from rest of patients. 4 of these 7 cultures, which did not change parasite numbers under neutralising conditions showed parasite growth upto last well, so titration of splenic biopsies over twelve wells would bring some insight into this. While 14.8% cultures which showed decrease in number of parasite, the end point of parasite growth differed by one or two wells only. Previously, we showed that neutralization of IFN-γ alone would cause increase in number of viable splenic amastigotes [7], whereas individual blocking of TNF-α does not altered the parasite load in comparison to control treated cultures [14]. So further to understand if combination of these cytokines would work better, we tested direct impact of IFN-γ and TNF-α blockade in splenic cultures. But here, in contrast to expectations we did not observed any additive effect of blocking these cytokines in conjunction, as it did not induced more parasite survival than that could be accomplished by blocking IFN-γ [7] or TNF-α [14] alone. Since we did not observed any additional effect of combine blockade, this would imply that increase in parasite survival can be effect of IFN-γ only and combination of these two cytokines may be inactive in this respect. One potential interpretation could be that our short term splenic culture only allows us to see direct effects on parasite survival and modulation of those up or down stream molecules, targeting parasite growth may not be quickly detectable. However, in earlier studies TNF-α has been identified to synergize with IFN-γ for production of NO_2_^−^ [23], which mediated arginine dependent parasite killing in murine model of Leishmania infection [24, 25].

Moreover, estimation of cytokine concentrations during 72hr revealed elevated IL-4 levels while no change in IL-10 production in anti-IFN-γ and anti-TNF-α treated cultures. Using the same short term splenic culture previously we showed that individual blockade of IFN-γ or TNF-α mediate no changes in IL-10 production [7, 14]. In present study we found combine blockade of these two cytokines are not sufficient to induce significant release of IL-10 in comparison to control. It is evident that regulatory cytokine IL-10 is secreted by wide range of cells [26]. Control of IL-10 secretion can be complex and/or multifactorial [27], so potentially IFN-γ and TNF-α are not directly involved in its coordination. Furthermore, combine neutralization of IFN-γ and TNF-α enabled significantly enhanced production of IL-4 in cultures when compared with control treated splenic biopsies. IFN-γ is recognized to inhibit IL-4 production in various systems [28, 29]. These two pro-inflammatory cytokines might be connected to a network that regulates IL-4 production but imparts no direct effect on IL-10 production. In experimental model of *L*. *major* infection IL-4 has been recognized to promote generation of protective immunity [30], IL-4 could bring this effect by enhancing expression of MHCII on macrophages and mediate their activation to increase parasite killing [31]. Susceptibility to infection was found to be IL-4 independent in BALB/c mice [32]. Importantly, previous studies showed that IL-4 does not exacerbate disease and even may play beneficial role against *L*. *donovani* infection [33]. IL-4 deficient mice show increased parasite load with retarded granuloma maturation and anti-leishmanial activity [34]. Endogenous IL-4 has been shown to play an important role in effective anti-leishmanial chemotherapy through maintainance of IFN-γ production following therapy [33]. However, IL-10 inhibits production of pro-inflammatory cytokines from macrophages and increases the parasite persistence [35]. In experimental VL inhibition of IL-10 signaling enhances Th1 responses and parasite killing [36], suggesting IL-10 as an appropriate target for therapeutic inhibition in VL [4]. Previous study from our laboratory also demonstrated that IL-10 neutralisation promotes parasite killing in VL patients, providing direct support for targeting IL-10 as a therapy [20].

Our data identifies CD4^+^T cells and CD14^+^ cells as the cellular source of IFN-γ and TNF-α that promoted killing of amastigotes in splenic aspirate cells. Further, we understand that one of the imperative approaches to better demonstrate the direct and combine impact of IFN-γ and TNF-α blockade on parasite burden using short term splenic cultures would be to compare their separate, individual blockade along with combined and control neutralization. But unfortunately we have confined access to human splenic aspirates and we were not able to further divide available splenic material into various subsets for comparative culture assay. Considering ambiguous evidences about the role of IL-4 in VL [33, 37], it remains an outstanding question to assess and confirm the biological role of cytokine IL-4 in target organ of VL. Further study is necessary focused to understand the therapeutic implications of IL-4 in human VL.

## Conclusions

Altogether, our results indicate that endogenous IFN-γ produced by splenic CD4^+^ T cells and TNF-α produced by CD14^+^ cells are essential component for host defense in VL spleen, but has no direct additive impact on parasite replication and IL-10 production. These findings further extend our understandings of how IFN-γ and TNF-α affects the production of type II cytokine IL-4. However our data does not support the idea of directly using combination of these cytokines for therapeutic purposes in VL. Nevertheless targeting these cytokines as an adjuvant therapy in patients with exaggerated inflammatory responses can have favourable outcomes.
